# Combined simultaneous multislice bSSFP and compressed sensing for first-pass myocardial perfusion at 1.5 T with high spatial resolution and coverage

**DOI:** 10.1002/mrm.28345

**Published:** 2020-06-12

**Authors:** Sarah McElroy, Giulio Ferrazzi, Muhummad Sohaib Nazir, Karl P. Kunze, Radhouene Neji, Peter Speier, Daniel Stäb, Christoph Forman, Reza Razavi, Amedeo Chiribiri, Sébastien Roujol

**Affiliations:** 1 School of Biomedical Engineering and Imaging Sciences, Faculty of Life Sciences and Medicine, King’s College London, London, United Kingdom; 2 MR Research Collaborations, Siemens Healthcare Limited, Frimley, United Kingdom; 3 Magnetic Resonance, Siemens Healthcare GmbH, Erlangen, Germany; 4 MR Research Collaborations, Siemens Healthcare Pty Ltd, Melbourne, Australia

**Keywords:** compressed sensing, myocardial perfusion, simultaneous multislice

## Abstract

**Purpose:**

To implement and evaluate a pseudorandom undersampling scheme for combined simultaneous multislice (SMS) balanced SSFP (bSSFP) and compressed-sensing (CS) reconstruction to enable myocardial perfusion imaging with high spatial resolution and coverage at 1.5 T.

**Methods:**

A prospective pseudorandom undersampling scheme that is compatible with SMS-bSSFP phase-cycling requirements and CS was developed. The SMS-bSSFP CS with pseudorandom and linear undersampling schemes were compared in a phantom. A high-resolution (1.4 × 1.4 mm^2^) six-slice SMS-bSSFP CS perfusion sequence was compared with a conventional (1.9 × 1.9 mm^2^) three-slice sequence in 10 patients. Qualitative assessment of image quality, perceived SNR, and number of diagnostic segments and quantitative measurements of sharpness, upslope index, and contrast ratio were performed.

**Results:**

In phantom experiments, pseudorandom undersampling resulted in residual artifact (RMS error) reduction by a factor of 7 compared with linear undersampling. In vivo, the proposed sequence demonstrated higher perceived SNR (2.9 ± 0.3 vs. 2.2 ± 0.6, *P* = .04), improved sharpness (0.35 ± 0.03 vs. 0.32 ± 0.05, *P* = .01), and a higher number of diagnostic segments (100% vs. 94%, *P* = .03) compared with the conventional sequence. There were no significant differences between the sequences in terms of image quality (2.5 ± 0.4 vs. 2.8 ± 0.2, *P* = .08), upslope index (0.11 ± 0.02 vs. 0.10 ± 0.01, *P* = .3), or contrast ratio (3.28 ± 0.35 vs. 3.36 ± 0.43, *P* = .7).

**Conclusion:**

A pseudorandom k-space undersampling compatible with SMS-bSSFP and CS reconstruction has been developed and enables cardiac MR perfusion imaging with increased spatial resolution and myocardial coverage, increased number of diagnostic segments and perceived SNR, and no difference in image quality, upslope index, and contrast ratio.

## 1 Introduction

Cardiac MR (CMR) perfusion imaging is an established technique used for the diagnosis of coronary artery disease and is recommended for ischemia assessment by current guidelines in the United States and Europe.^1,2^ A variety of techniques have been proposed for CMR perfusion imaging and current guidelines recommend the use of saturation recovery dynamic imaging with at least three short axis slices per heartbeat and in-plane resolution greater than 3 × 3 mm^2^.^3^ The technique can be applied with a balanced SSFP (bSSFP), FLASH, or hybrid EPI readout, with bSSFP providing an advantage in terms of SNR and contrast-to-noise ratio.^4,5^

Achieving high spatial resolution and spatial coverage may be valuable to increase confidence in the detection of perfusion defects in contrast-enhanced CMR perfusion. Increased spatial coverage is desirable to ensure that all myocardial perfusion territories can be assessed for perfusion defects and to improve the assessment of total ischemic burden,^6^ which has shown high prognostic value using nuclear myocardial perfusion imaging.^7^ Likewise, increased spatial resolution offers a number of advantages, including reduced dark rim artifact, which can mimic endocardial perfusion defects,^8^ and improved assessment of transmural perfusion gradients, which has been shown to predict hemodynamically significant coronary artery disease.^9^ Assessment of transmural perfusion gradients benefits from high spatial resolution,^9^ especially in patients with a thin left ventricular wall (eg, due to dilated cardiomyopathy or transmural myocardial infarction^10^).

Two-dimensional imaging using standard parallel imaging techniques is the most commonly used approach for CMR perfusion imaging but is associated with limited spatial coverage (usually 3-4 short-axis slices per heartbeat) and limited spatial resolution (2-3 × 2-3 mm^2^). Three-dimensional acquisition techniques have been developed to enable CMR perfusion with full heart coverage, but are also associated with limited in-plane spatial resolution (>2 × 2 mm^2^) due to the requirement to minimize cardiac motion using a sufficiently short readout duration.^11,12^ Alternatively, higher spatial resolution can be achieved with advanced acceleration schemes, such as *k-t* techniques^13–16^ or compressed sensing (CS).^17–19^ Compressed sensing enables high acceleration by exploiting the sparsity of MR images, usually in some transform domain (eg, wavelet, finite differences). It does not require additional training data (as for *k-t* techniques), but requires that artifacts caused by k-space undersampling be incoherent in the sparse transform domain, which is commonly achieved using random undersampling schemes.

Simultaneous multislice (SMS) is a 2D imaging technique that uses multiband RF pulses to excite multiple slices simultaneously.^20–23^ This can enable imaging of multiple slices within the same time as a conventional single-slice acquisition, resulting in increased spatial coverage without degrading spatial resolution. The SMS technique can be combined with controlled aliasing in parallel imaging results in higher acceleration (CAIPIRINHA) encoding.^24^ By providing the simultaneously excited slices with individual RF phase cycles, SMS-CAIPIRINHA introduces a slice-specific phase modulation of k-space that results in a shift of the slices with respect to each other in the FOV and improves parallel imaging-based image reconstruction. The integration of SMS-CAIPIRINHA with a FLASH readout is straightforward and has been applied previously to CMR perfusion imaging.^25–30^

Combining SMS-CAIPIRINHA with a bSSFP readout is more challenging, as in addition to realizing the aforementioned slice-specific phase modulation of k-space, the bSSFP steady state needs to be maintained and the frequency response centered with respect to the water peak, to minimize undesired off-resonance effects. To that end, CAIPIRINHA-compatible bSSFP phase-cycling schemes^31^ can be used in addition to the recently presented gradient-controlled local Larmour adjustment (GC-LOLA) concept.^32^ This SMS-bSSFP technique has been applied to CMR perfusion imaging, resulting in increased spatial coverage and matched resolution compared with a conventional 2D protocol.^33^

Combining this technique with CS to enable higher acceleration has the potential to simultaneously provide high spatial resolution and coverage for CMR perfusion. Current SMS-bSSFP techniques use a linear undersampling scheme^33^ that is suboptimal for CS reconstruction, as it generates coherent artifacts, limiting the achievable acceleration. Pseudorandom undersampling schemes generate a more incoherent aliasing pattern, but for application to SMS-bSSFP the pseudorandom undersampling pattern must obey the requirements for a slice-specific k-space phase modulation and RF phase cycling.

In this work, we sought to develop a pseudorandom undersampling scheme for SMS-bSSFP with CS reconstruction. We demonstrate the compatibility of the pseudorandom undersampling scheme with CS reconstruction through computation of the point spread function (PSF) and phantom studies. The high acceleration achievable using this framework is applied to a 1.5T SMS-bSSFP perfusion sequence with high spatial resolution (1.4 × 1.4 mm^2^) and high spatial coverage (six slices).

## 2 Theory

### 2.1 Radiofrequency phase-cycled SMS-CAIPIRINHA with bSSFP imaging

Radiofrequency phase-cycled SMS-CAIPIRINHA exploits the results of the Fourier shift theorem, which states that a phase ramp in k-space corresponds to a shift in image space.^24^ This is challenging to apply to bSSFP imaging, which usually requires 180° phase cycling of successive RF pulses to center the passband of the frequency response over the resonance frequency. In this study, we use the SMS-bSSFP solution proposed by Stäb et al, which applies a π/2 and 3π/2 k-space phase modulation to the first and second slice, respectively (ie, four phase cycling steps for each slice: 0, π/2, π, 3π/2 [slice 1] and 0, 3π/2, π, π/2 [slice 2]), resulting in a half-FOV shift between the two slices in the phase-encode direction.^31^ For a fully sampled k-space with linear encoding, this generates a phase cycle of π/2 (slice 1) and 3π/2 (slice 2) between subsequent RF pulses, which results in a slightly off-centered frequency response profile for both slices. To correct for this, GC-LOLA was used, which applies an additional gradient in the slice direction to realign the passband over the resonance frequency for both slices.^32^

### 2.2 Simultaneous multislice bSSFP with pseudorandom undersampling

To design a pseudorandom undersampling scheme that is compatible with the SMS-bSSFP with GC-LOLA solution outlined in the previous section, it is necessary to adhere to two constraints:

1. The phase imparted to each acquired k-space line must maintain the slice-specific k-space phase modulation (π/2 and 3π/2 for slices 1 and 2, respectively).
2. The phase of each subsequently applied RF pulse must obey the phase cycling scheme (also π/2 and 3π/2 for slices 1 and 2, respectively).

Therefore, the acquisition is constrained by which particular k-space indices are selected and the order in which they are acquired. In the case of a fully sampled k-space acquisition with linear encoding, both requirements are fulfilled. Conversely, if k-space indices are selected at random and acquired with the phase corresponding to their k-space index (ie, maintaining the k-space phase modulation), the RF phase cycling condition would not be satisfied.

**Figure 1:**
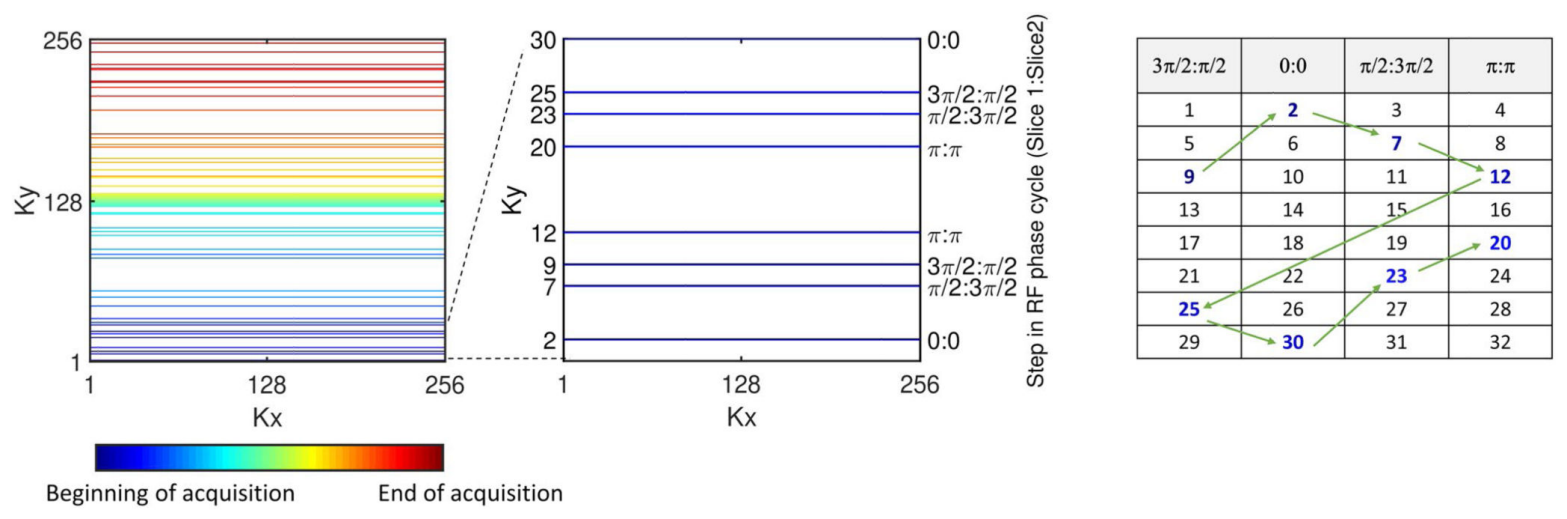
Proposed undersampling scheme illustrated for a multiband factor of 2. Left: Representation of full k-space with pseudorandom undersampling consistent with the requirements of a simultaneous multislice (SMS)–balanced SSFP (bSSFP) acquisition. Middle: Zoom of first 30 k-space lines. Right: Acquisition order of selected k-space indices. Shaded column headings correspond to the four steps in the RF phase cycle for SMS-bSSFP with multiband factor 2 (slice 1 phase:slice 2 phase). k-Space indices are binned according to their step in the RF phase cycle, and an equal number of indices are randomly selected from each bin. Colored numbers represent pseudorandomly selected k-space phase-encode indices. Order of acquisition is cycled through each bin sequentially (green arrows) to maintain the SMS-bSSFP phase-cycling requirement

In the current work, we have developed an algorithm to enable pseudorandom undersampling (Figure 1). The undersampled k-space includes a fully sampled central k-space region, while the upper and lower halves of peripheral k-space are pseudorandomly undersampled to achieve the desired acceleration. Thus, the number of randomly sampled peripheral k-space lines is calculated as the total number of lines divided by the acceleration factor, minus the number of fully sampled central k-space lines, with an equal number of k-space lines acquired in the lower and upper halves of k-space. To ensure that the chronological order of RF phase cycling is maintained throughout the acquisition, it is necessary to select an equal number of k-space indices corresponding to each step in the RF cycle (as for the fully sampled case). To achieve this, all k-space line indices are binned into groups corresponding to their k-space phase modulation/RF phase cycle step (in the case of multiband 2, there are four possible steps, so four groups in total). The number of k-space indices randomly selected from each bin are evenly distributed across the four bins, while ensuring that the transition between central and peripheral k-space lines maintains the RF phase cycle. This method is applied to upper and lower peripheries of k-space separately, so that jumps between upper and lower k-space are avoided. The order of the acquisition is as follows: The selected lower peripheral k-space lines are acquired from each bin sequentially, followed by the fully sampled central k-space lines, followed by the selected upper peripheral k-space lines, again from each bin sequentially, thereby maintaining the RF phase cycle. The process is applied to each dynamic independently to achieve temporal incoherence. Figure 1 presents an example trajectory produced using the described algorithm. The algorithm is described by the following pseudocode:

for each dynamic

{

for each peripheral region (ie, lower and upper)

{

- Sort lines from region into four bins (according to their phase-cycle step)

for each of the four bins

{

- Select randomly N different ky line indices from the current bin
- Sort selected ky line indices for the current bin in ascending order
- Store selected ky line indices for current bin and peripheral region

}

}

- Add randomly selected lower periphery k-space indices to the trajectory, cycling through the four bins in turn
- Add fully sampled center lines to trajectory in ascending order
- Add randomly selected upper periphery k-space indices to trajectory, cycling through the four bins in turn

}

### 2.3 Compressed-sensing reconstruction

This SMS-bSSFP sequence with pseudorandom undersampling was combined with an inline CS reconstruction previously described.^34^ Briefly, it uses a nonlinear iterative reconstruction framework with L1 regularization in the spatial and temporal wavelet domains:

(1) $$\begin{array}{ll} {\left\{x_{t}\right\}}_{t-1,...T} & =\underset{\left\{x_{t}\right\}}{\arg\;\min}\;\sum_{t=1}^{T}\left({\left\|A_{t}x_{t}-y_{t}\right\|}_{2}^{2}+\lambda_{\omega }{\left\|W_{\omega }x_{t}\right\|}_{1}\right)\\ \; & +\lambda_{\tau }{\left\|W_{\tau }\left\{x_{`1}^{T},...\;x_{T}^{T}\right\}\right\|}_{1}. \end{array}$$

For each time point *t*, *x_t_* represents the estimated fully sampled image; *A_t_* incorporates the acquired sampling pattern, the Fourier transform, and coil sensitivities; *y_t_* represents the acquired undersampled data; and *W_ω_* and *W_τ_* represent the spatial and temporal wavelet transforms, respectively, whereas λ_ω_ and *λ_τ_* are regularization parameters. The fast iterative shrinkage-thresholding algorithm^35^ is used to perform the iterative reconstruction, using the Chambolle-Pock algorithm^36^ for calculation of the proximal operator of the L1 terms.

### 2.4 Implementation

Spatial and temporal regularization parameters of the CS reconstruction were kept fixed at 0.01 and 0.05, respectively, to maximize the image quality, as optimized for SMS-bSSFP perfusion imaging in a previous study^33^ and in a pilot study in 1 patient using the proposed approach. The total number of iterations was fixed at 60. A “lean” implementation of CAIPIRINHA was used for slice separation as proposed in Stäb et al.^37^ This implementation uses phase oversampling of 100% (in the case of multiband 2 acceleration), so that slice separation can be readily achieved along the phase-encoding direction, as the (shifted) slices are reconstructed onto the oversampled FOV.^21^

## 3 Methods

All imaging studies were performed at 1.5 T (MAGNETOM Aera; Siemens Healthcare, Erlangen, Germany) (VE11C software) with a 32-channel spine array and an 18-element body coil.

### 3.1 Point spread function analysis

To investigate potential remaining coherences, the PSF in x-f space was computed by taking the Fourier transform of the k-space sampling lattice, along the temporal and phase-encoding dimensions. This was performed for three different undersampling schemes: a purely random undersampling scheme, the pseudorandom undersampling scheme derived using the algorithm presented in this study, and a linear (TGRAPPA) undersampling scheme. All schemes were modeled for dual-slice excitation with the SMS-bSSFP slice-specific k-space phase modulations (slice 1: π/2 and slice 2: 3π/2). To avoid smearing in the calculated PSFs along the phase-encoding direction, our pseudorandom undersampling scheme was modeled with no fully sampled center k-space lines.

### 3.2 Phantom studies

#### 3.2.1 Comparison of pseudorandom and linear undersampling schemes

A phantom with multiple vials representative of typical precontrast and postcontrast myocardial T_1_ and T_2_ values^38^ was scanned with a prototype SMS-bSSFP perfusion sequence using our proposed pseudorandom undersampling scheme (SMS-CS) and a linear undersampling scheme (SMS-TGRAPPA) at increasing acceleration factors: 3-7 (linear) and 3-13 (pseudorandom), where the total acceleration factor is calculated as the product of the slice-acceleration factor and in-plane acceleration factor. Other acquisition parameters were as follows: TR/TE/α = 3.5 ms/1.5 ms/50°, saturation time = 240 ms for 1.5-times in-plane acceleration factor and 165 ms for in-plane acceleration factors of 2.5 or higher, FOV = 150 × 150 mm^2^, resolution = 0.94 × 0.94 mm^2^, slice thickness = 10 mm, bandwidth = 945 Hz/Px, and number of dynamics = 20. All phantom images were reconstructed inline on the scanner using the reconstruction algorithm described in section 2. Images acquired with in-plane acceleration factor greater than 2.5 were subtracted from the corresponding images acquired with an inplane acceleration factor of 2.5. The normalized RMS error (NRMSE) was calculated for each frame of phantom series:

$$NRMSE=\frac{1}{x_{max}-x_{min}}\sqrt{\frac{\sum_{i=1}^{N}{\left(x_{i}-{\hat{x}}_{i}\right)}^{2}}{N}}$$

where *i* represents pixel number; *N* is the total number of pixels; *x* is the reference image at low acceleration; *x_max_* and *x_min_* are the maximum and minimum signal intensity values of the reference image; and $\hat{x}$ is the reconstructed image at high acceleration. The mean NRMSE was then calculated as the average across the 20 frames.

#### 3.2.2 Evaluation of reconstructed spatial resolution

A resolution phantom was scanned to compare the sharpness of phantom structures using the proposed SMS-CS sequence against a fully sampled acquisition. The SMS-CS sequence used a total acceleration factor of 11 to achieve high spatial resolution while minimizing the presence of artefacts. This enabled a spatial resolution of 1.4 × 1.4 mm^2^ for a FOV of 360 × 360 mm^2^, which is comparable to previous high-resolution CMR perfusion studies.^39–41^ Phantom images were also acquired with a pixel size of 1.5 × 1.5 mm^2^ (corresponding to a FOV of 380 × 380 mm^2^). Fully sampled phantom images were acquired with an in-plane spatial resolution varying between 1.4 × 1.4 mm^2^ and 1.9 × 1.9 mm^2^ in increments of 0.1 mm. Fully sampled acquisitions were segmented to ensure the saturation time was consistent between all acquisitions.

Sharpness was measured as previously described.^42,43^ This method calculates the distance for the signal-intensity profile across a high-contrast boundary to drop from 80% to 20% of the signal range. A sharpness index is then calculated as the reciprocal of this distance. Four signal intensity profiles were drawn at the center of the two horizontal and two vertical edges of the square block contained within the phantom, and the average sharpness index across all profiles was calculated. The sharpness index measured on images acquired with the proposed sequence was compared with those measured on fully sampled images.

### 3.3 In vivo evaluation

Ten patients (8 male, 2 female, mean age 40 ± 16 years) referred for a clinical contrast-enhanced CMR scan were prospectively recruited for the study. This study was approved by the National Research Ethics Service (15/NS/0030), and written informed consent was obtained from all patients for the scan and for inclusion in this study. All patients underwent two rest perfusion scans using a reference sequence (conventional) and the proposed sequence (SMS-CS). All patients were asked to hold their breath for the duration of the first pass of the contrast agent. The conventional sequence is a three-slice (single-band) saturation-recovery bSSFP sequence with standard GRAPPA reconstruction, as optimized for clinical practice. The proposed sequence is a six-slice, high-resolution SMS-bSSFP sequence with CS reconstruction. Acquisition parameters were as follows: FOV = 360 × 360 mm^2^; TR/TE/flip angle = 2.5 ms/1.04 ms/50° (conventional) and 2.9 ms/1.24 ms/50° (SMS-CS); saturation time = 94 ms; pixel size = 1.9 × 1.9 mm^2^ (conventional) and 1.4 × 1.4 mm^2^ (SMS-CS); slice thickness = 10 mm; in-plane acceleration = 3 (conventional) and 5.5 (SMS-CS); multiband acceleration factor = 2 (SMS-CS only); readout duration per slice/slice group = 156 ms (conventional) and 137 ms (SMS-CS); acquisition duration for all slices = 564 ms (conventional) and 540 ms (SMS-CS); and bandwidth = 1302 Hz/Px. This resulted in a total acceleration factor of 3 and 11 for the conventional and proposed sequences, respectively. A total dose of 0.075 mmol/kg of gadobutrol (Gadovist; Bayer, Berlin, Germany) was administered for each perfusion protocol. The two sequences were performed in a random order separated by a minimum time interval of 10 minutes to allow for wash-out of the contrast agent. Both sequences were planned on the systolic phase of two-, three-, and four-chamber cine images to ensure coverage of the base, mid, and apical slices. After prescribing the position of the slices in the base–apex direction, the slice orientation was rotated in the short-axis plane so that the phase-encode axis was aligned with the shortest bodily dimension. The whole-body specific absorption rate was recorded for each sequence and for all patients.

#### 3.3.1 Qualitative assessment

The first pass of each acquisition, acquired during a breath-hold, was assessed qualitatively in consensus by 2 expert readers (A.C. and S.N.) with more than 10 and 5 years of CMR experience, respectively. The CMR readers were blinded from the patient information but not from the acquisition method, as images had unambiguous different properties (especially in terms of SNR). Data visualization was performed using Osirix software (OsiriX Foundation, Geneva, Switzerland). Image quality was assessed for each slice (3 = excellent, 2 = minor artifact but not limiting diagnosis, 1 = major artifact but not limiting diagnosis, 0 = poor image quality and nondiagnostic), excluding any images acquired outside the heart. The frequency of each image quality score was normalized to the total number of slices included in the assessment of each sequence. Additionally, an overall perceived SNR score was determined for each sequence (3 = high SNR, 2 = minor noise level but not limiting diagnosis, 1 = major noise level but not limiting diagnosis, and 0 = poor SNR resulting in nondiagnostic image quality). Finally, the diagnostic value of each scan was assessed on a segmental basis. Each American Heart Association (AHA) myocardial segment^44^ was scored as diagnostic or nondiagnostic. If the base/apex were not included in the acquired slices, the segments from these slices were recorded as nondiagnostic.

#### 3.3.2 Quantitative assessment

**Figure 2:**
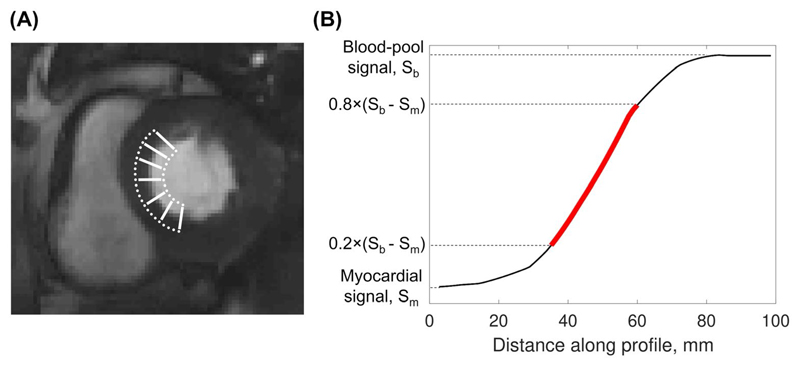
Illustration of sharpness measurements in vivo. A, Typical locations of line profiles drawn across the septal blood-myocardium boundary. B, Red line represents the slope between 20% and 80% of the difference between myocardial and peak blood-pool signal intensity (S_b_-S_m_). The sharpness index is calculated as 1/d, where d is the distance over which the signal-intensity profile increases from 20% to 80% of the signal range

Quantitative assessment was performed to compare image sharpness, contrast ratio, and upslope index between the conventional and proposed techniques. The sharpness index was determined as for the phantom study, using the septal blood–myocardium boundary at peak enhancement of the left ventricular blood pool as a high-contrast interface. Because cardiac motion and slice position can affect the sharpness of the blood–myocardium boundary, the sharpness measurements were performed on three slices for each sequence: For each of the slices acquired with the conventional sequence, the slice closest in position and acquired in the same cardiac phase was selected from the SMS-CS data set. For each slice, a curve with multiple closely spaced points was drawn at either side of the septal blood–myocardium interface. A profile was generated between each point on the myocardial curve, and the closest corresponding point on the blood pool curve and the sharpness index was computed (Figure 2). The average sharpness index across all profiles was then calculated. Upslope index and contrast ratio were both measured on a midventricular slice. Upslope index was measured as the ratio of maximum gradient of the myocardial time-intensity curve and the maximum gradient of the left ventricular blood pool time-intensity curve.^45^ The gradient was computed using a four-point linear fit, with a sliding window. Contrast ratio was measured as the ratio of the peak myocardial signal intensity and the peak blood pool signal intensity.^33^

#### 3.3.3 Statistical analysis

All statistical analyses were performed using statistical software (SPSS Version 25; IBM, Armonk, NY). Results of the qualitative assessment are expressed as mean ± SD. The Wilcoxon signed-rank test was used to compare image quality and perceived SNR scores between the two sequences. The McNemar test was used to compare the number of diagnostic segments between the sequences. Sharpness, up-slope ratio, and contrast-ratio measurements were compared using paired *t*-tests. A *P*-value < .05 was considered significant.

## 4 Results

### 4.1 Point spread function

**Figure 3:**
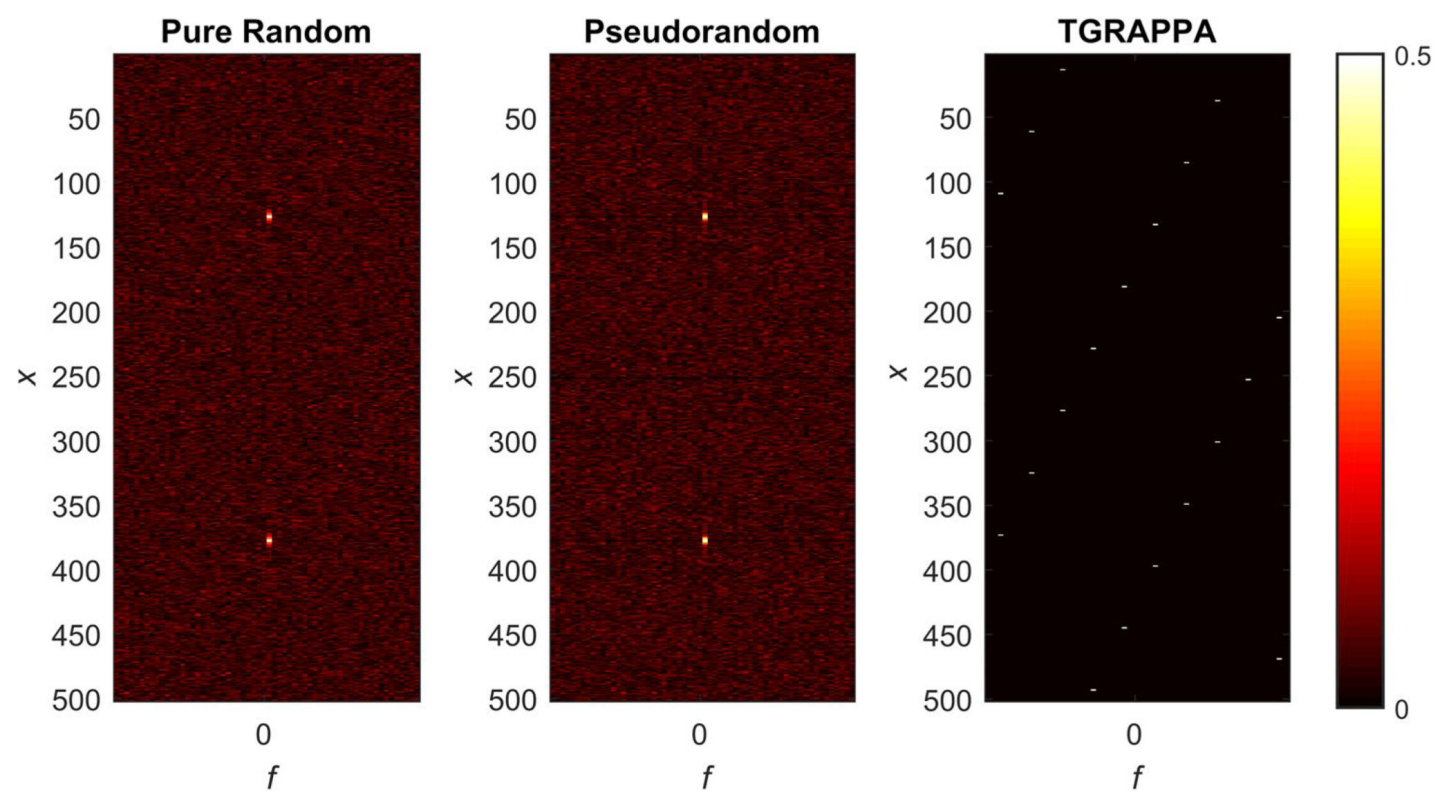
Point spread function in x-f space of a multiband factor 2 acquisition using a purely random undersampling scheme (left), the proposed pseudorandom undersampling scheme (middle), and a TGRAPPA linear undersampling scheme (right). The phase-cycling scheme used the results in a spatial displacement of slice 1 and slice 2 by +1/4 FOV and -1/4 FOV in the phase-encode dimension, respectively

The PSF in x-f space is presented in Figure 3 for the purely random undersampling scheme (left), the proposed pseudorandom undersampling scheme (middle), and finally, a linear (TGRAPPA) undersampling scheme (right). Two main peaks representing the two simultaneously excited slices are visualized for the purely random and pseudorandom undersampling schemes. A noise-like pattern was observed over the rest of the PSF for both schemes, maximizing incoherent aliasing artifacts as required for CS reconstruction. Conversely, the PSF of the linear undersampling scheme contains multiple peaks promoting coherent aliasing artefacts.

### 4.2 Phantom studies

#### 4.2.1 Comparison of pseudorandom and linear undersampling schemes

**Figure 4:**
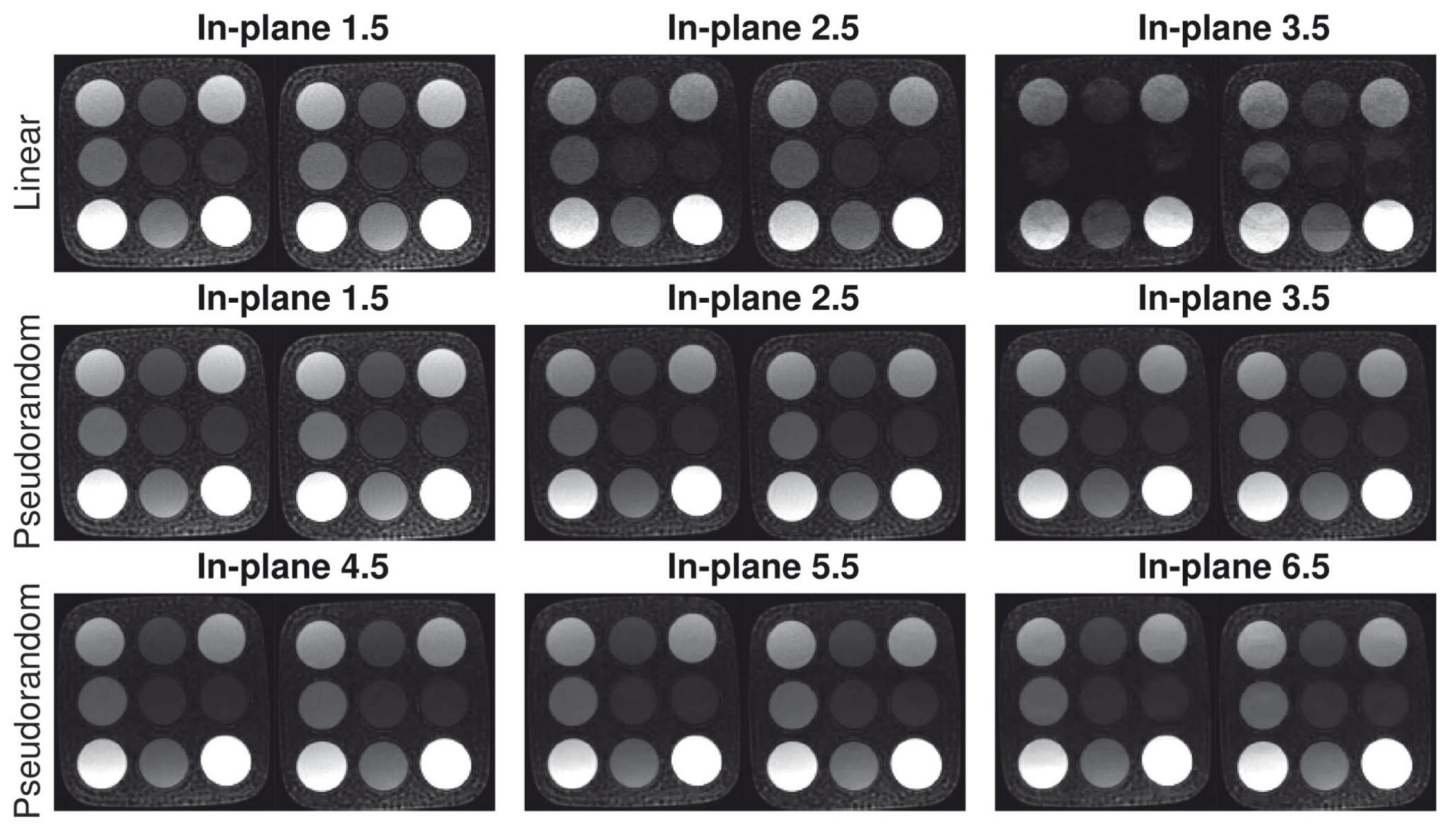
Phantom study using SMS-CS and SMS-TGRAPPA with a slice-acceleration factor of 2 (SMS 2), in-plane resolution 0.9 × 0.9 mm, and different in-plane acceleration factors. Top row: SMS-TGRAPPA with a total acceleration factor of 3, 5, and 7 (in-plane acceleration = 1.5, 2.5, and 3.5; slice acceleration = 2). Middle row: SMS-CS with a total acceleration factor of 3, 5, and 7 (in-plane acceleration = 1.5, 2.5, and 3.5; slice acceleration = 2). Bottom row: SMS-CS with a total acceleration factor of 9, 11, and 13 (in-plane acceleration = 4.5, 5.5, and 6.5; slice acceleration = 2). The two acquired slices are shown for each scan

Reconstructed images from the phantom study comparing SMS-CS and SMS-TGRAPPA with iterative reconstruction are presented in Figure 4. At a high acceleration factor of 7, SMS-CS resulted in reduced residual artifacts (NRMSE = 0.74%) compared with SMS-TGRAPA (NRMSE = 5.77%). For all higher-acceleration factors,^9–13^ SMS-CS resulted in a NRMSE of less than 2% (see subtraction images and their corresponding NRMSE in Supporting Information Figure S1). The SMS-CS technique results in an accurate and temporally stable reconstruction up to a total acceleration of 11, with minor artifacts appearing at higher acceleration factors.

#### 4.2.2 Evaluation of reconstructed spatial resolution

Sharpness measurements for the proposed SMS-CS sequence and for fully sampled acquisitions are presented in Table 1. Higher resolution resulted in an increased sharpness index for both the SMS-CS and fully sampled acquisitions. The sharpness index for SMS-CS is comparable to a fully sampled acquisition with the same matrix size.

### 4.3 In vivo evaluation

**Figure 5:**
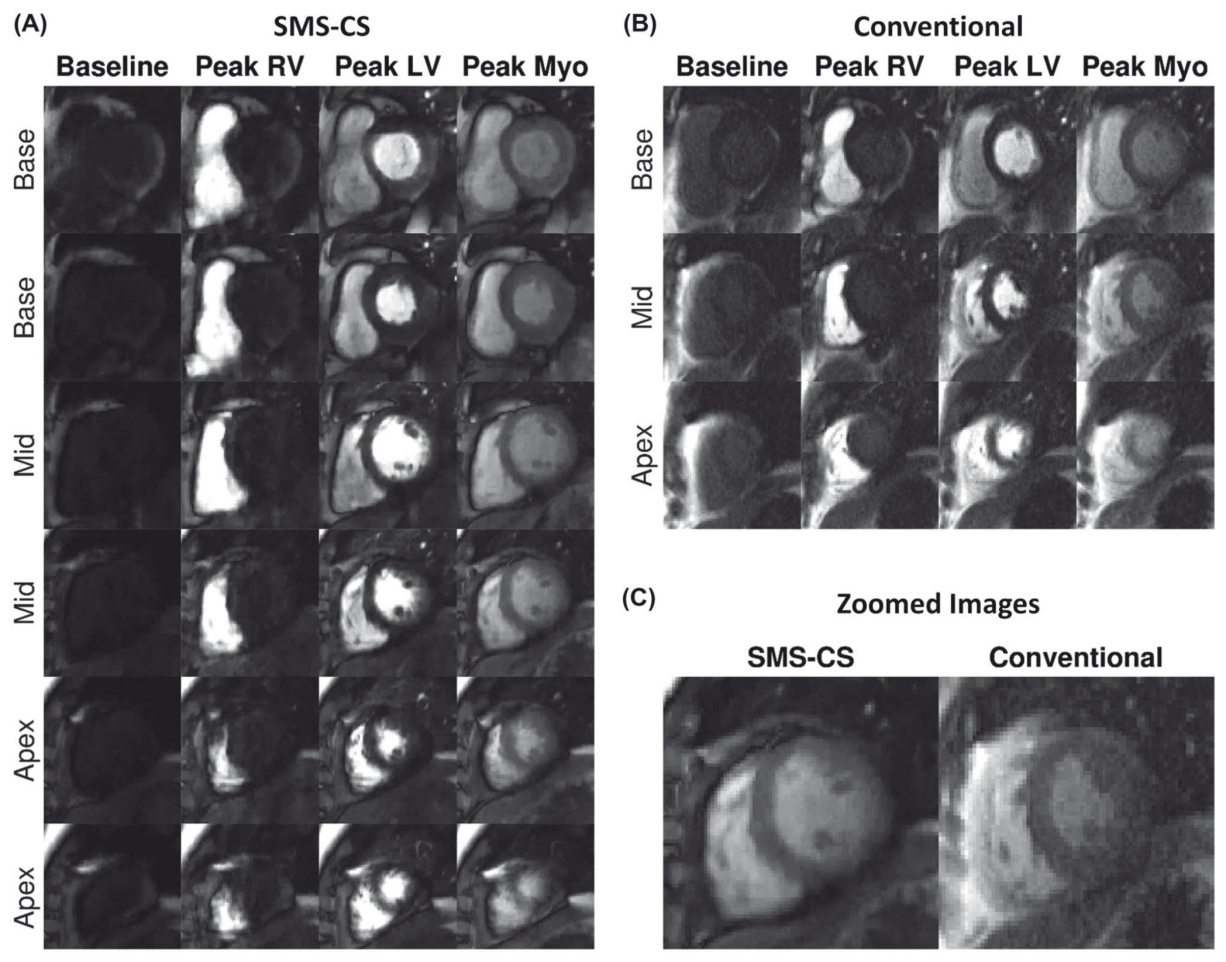
In vivo evaluation in a patient. A, The SMS-CS perfusion images with high spatial resolution (1.4 × 1.4 mm^2^) and high spatial coverage (six slices). B, Conventional three-slice bSSFP perfusion acquisition with in-plane resolution of 1.9 × 1.9 mm^2^ and GRAPPA reconstruction. C, Zoomed image acquired at peak myocardial signal enhancement with SMS-CS and conventional acquisitions. Average image-quality scores were equivalent (3 = excellent) for both perfusion sequences acquired in this patient. Abbreviations: LV, left ventricular; Myo, myocardial; and RV, right ventricular

**Figure 6:**
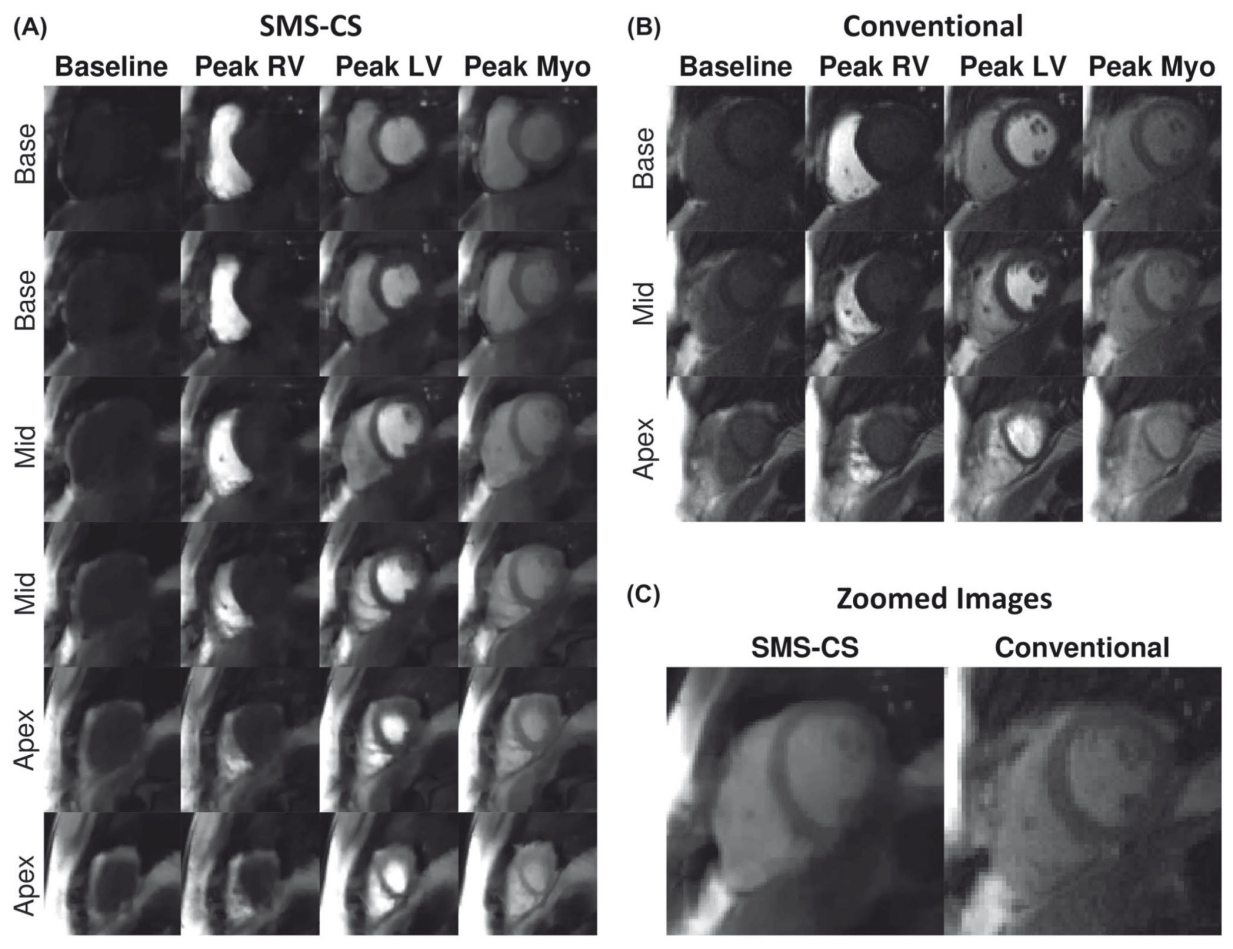
In vivo evaluation in a patient with a poor breath-hold. A, The SMS-CS perfusion images with high spatial resolution (1.4 × 1.4 mm^2^) and high spatial coverage (six slices). B, Conventional three-slice bSSFP perfusion acquisition with in-plane resolution of 1.9 × 1.9 mm^2^ and GRAPPA reconstruction. C, Zoomed image acquired at peak myocardial signal enhancement with SMS-CS and conventional acquisitions. Increased level of blurring artifacts can be observed in the SMS-CS images due to the poor breath-hold. The average subjective image-quality score for the SMS-CS sequence was 1.7, compared with 2.7 for the conventional acquisition; however, all 16 AHA segments were still deemed to be of diagnostic quality

Figure 5 shows example images acquired in a patient with the conventional sequence and the SMS-CS sequence. Excellent image quality was obtained with both sequences, whereas higher perceived SNR, higher spatial resolution, and doubled slice coverage were achieved using the proposed SMS-CS. Figure 6 shows the worst case obtained using the proposed sequence. In this case the patient did not perform a stable breath-hold during the first pass of the perfusion acquisition, resulting in increased image blurring with the proposed sequence. The average subjective image quality score for this sequence was 1.7, compared with 2.7 for the conventional acquisition; however, all 16 AHA segments were still deemed to be of diagnostic quality.

**Figure 7:**
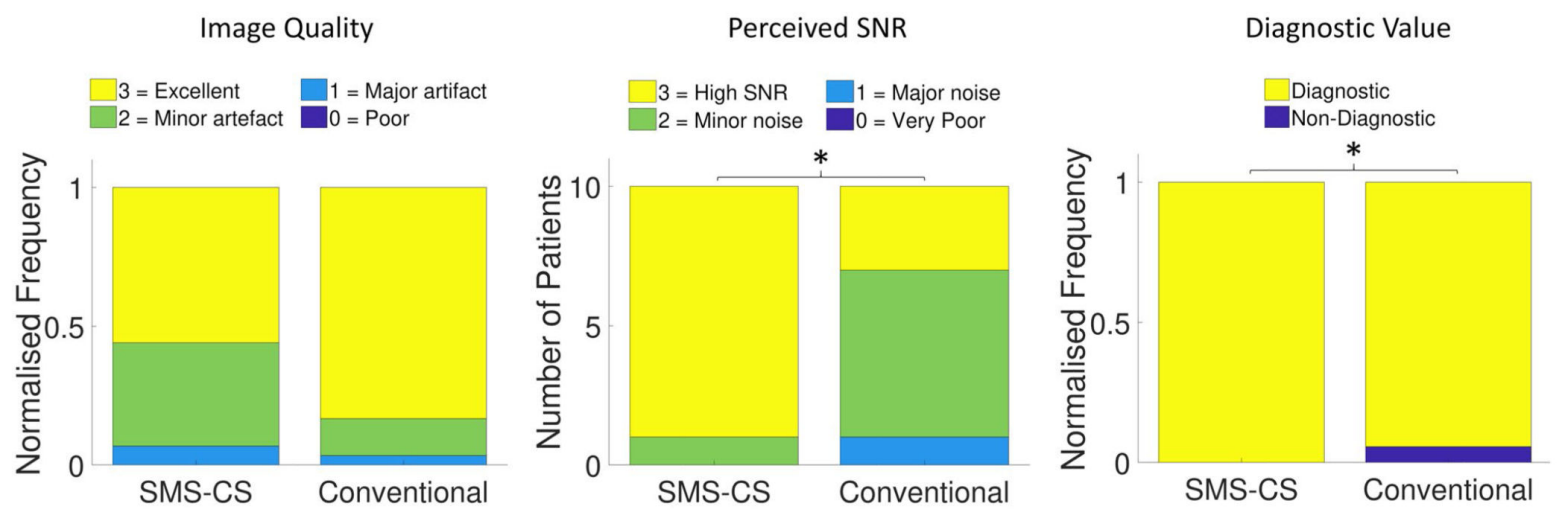
Results from image-quality assessment in 10 patients scored in consensus by 2 expert readers. (A) Normalized frequency distribution of image-quality scores for each sequence (*P* = .08). (B) Distribution of perceived SNR scores for each sequence. Perceived SNR is higher for the SMS-CS sequence than for the conventional sequence (*P* = .04). (C) Normalized frequency distribution of the number of diagnostic segments using each sequence. Number of diagnostic segments was higher using the SMS-CS sequence (*P* = .03). *A significant difference between conventional and SMS-CS results (*P* < .05)

Across all patients, there was no significant difference between the SMS-CS and conventional sequences in terms of mean image quality score (2.5 ± 0.4 vs. 2.8 ± 0.2, *P* = .08; Figure 7A). The SMS-CS technique yielded a higher perceived SNR (2.9 ± 0.3 vs. 2.2 ± 0.6, *P* = .04; Figure 7B) and a higher percentage of diagnostic segments (100% vs. 94%, *P* = .03; Figure 7C) than the conventional sequence. Average sharpness was higher with the proposed sequence than with the conventional sequence (0.35 ± 0.03 vs. 0.32 ± 0.05, *P* = .01). There were no significant differences between SMS-CS and conventional sequences for upslope index (0.11 ± 0.02 vs. 0.10 ± 0.01, *P* = .3) or contrast ratio (3.28 ± 0.35 vs. 3.36 ± 0.43, *P* = .7). Energy deposition for the SMS-CS sequence (7500 J) was about 13% higher than the conventional sequence (6651 J). The whole-body specific absorption rate for both sequences was below the normal mode limit (2 W/Kg) for all patients.

## 5 Discussion

In this work we developed a prospective pseudorandom undersampling pattern meeting the requirements of SMS-bSSFP for myocardial perfusion imaging with a CS reconstruction. This pseudorandom undersampling scheme enabled much higher acceleration in a phantom compared with a conventional linear undersampling scheme. The higher achievable acceleration was invested in increased spatial resolution for high-spatial-coverage SMS-bSSFP perfusion imaging. The proposed sequence demonstrated higher perceived SNR, a higher number of diagnostic segments, and similar image quality compared with a conventional acquisition, with doubled spatial coverage and increased spatial resolution. Furthermore, quantitative measurements showed increased sharpness and no significant differences in measurements of contrast ratio or upslope index.

The trade-off between spatial coverage and spatial resolution in CMR perfusion exists due to a combination of the physiologically imposed limits on the acquisition window and the high temporal resolution required. The combination of SMS with CS reconstruction offers a double-barreled approach to overcome this trade-off. Previous studies have combined SMS with CS previously using spiral and radial trajectories with a FLASH readout scheme.^26–30^ To our knowledge, CS with pseudorandom undersampling has not yet been applied to SMS with a bSSFP readout. Although there is debate as to the optimal readout scheme for CMR perfusion imaging, it has been demonstrated that bSSFP has superior SNR and contrast-to-noise ratio properties compared with the FLASH and hybrid-EPI schemes.^4,5,46^ Furthermore, bSSFP is used widely in other areas of CMR imaging due to these advantageous properties. Therefore, there may be value in applying this highly accelerated approach to other CMR sequences such as cine and late-gadolinium enhancement imaging.

The CMR perfusion studies are typically evaluated using the AHA segmentation model.^44^ An additional benefit of increased spatial coverage compared with conventional acquisitions is the increased likelihood of achieving diagnostic image quality in all 16 AHA segments. For all patients in this study, it was possible to assess all 16 AHA segments for perfusion defects using the proposed SMS-CS sequence. However, for 1 patient, the prescription of the first slice of the conventional perfusion sequence was not sufficiently basal. The result of this was that assessment of 6/16 AHA segments was not possible in this patient, as it may lead to an underestimation of ischemic burden or a false-negative test with risk of future adverse cardiovascular events. While careful planning can mitigate this issue in most cases, the problem can also arise due to inconsistent breath-holding or patient motion between the images used for planning and the perfusion acquisition.

In the current work, the slice coverage was doubled using a multiband slice acceleration of 2. Full left ventricular coverage was therefore only achieved in 2 patients. Further slice acceleration (using a multiband factor of 3 or 4) could enable full left ventricular coverage in most patients, allowing for assessment of total ischemic burden as achieved using 3D techniques. It is noted that specific absorption rate limits are more likely to become an issue at these higher multiband factors, which could impose a limit on the achievable flip angle and resultant SNR.

In a previous SMS-bSSFP study,^33^ doubled slice coverage using linear undersampling was reported, which restricted the maximum achievable acceleration. Furthermore, the SMS-bSSFP with the GC-LOLA framework is restricted to particular acceleration factors when linear undersampling is used, to maintain the k-space phase modulation and RF phase-cycling requirements. The pseudorandom undersampling algorithm developed in the current study enabled unrestricted selection of undersampling factors.

The GC-LOLA technique used in this study substantially reduces the sensitivity of the original SMS-bSSFP sequence to off-resonance.^32^ With GC-LOLA, the off-resonance response is realigned to that of conventional bSSFP at the cost of slightly widened banding artifacts. However, no banding artifacts were observed across the myocardium in this study, which is consistent with our previous work^33^; therefore, band-widening did not affect the clinical assessment of the images.

The SMS-bSSFP sequence can also be achieved using blipped CAIPIRINHA encoding,^47,48^ which has some potential advantages over RF phase cycling–based CAIPIRINHA encoding: It removes some of the restrictions for pseudorandom undersampling, as the bSSFP phase cycling can be applied independently of the required k-space modulation, and it does not suffer from widened banding artifacts that are introduced by the GC-LOLA correction. However, blipped CAIPIRINHA may be more susceptible to eddy current artifacts,^49^ flow artifacts,^50^ and fat leakage artifacts.^47,51^ A head-to-head comparison of SMS-bSSFP with blipped and RF phase-cycled CAIPIRINHA would help to understand and elucidate these differences.

In this study we used a total acceleration of 11 in vivo (inplane acceleration 5.5 × slice acceleration 2). This enabled us to achieve an in-plane spatial resolution of 1.4 × 1.4 mm^2^, which is comparable to previous studies of high-resolution CMR.^39–41^ The achieved spatial resolution in vivo may be lower than the nominal resolution due to the confounding effects of cardiac motion, partial-volume effects. and the reconstruction framework that uses spatial and temporal regularization. However, our results indicate a higher sharpness index with the proposed sequence (nominal resolution = 1.4 × 1.4 mm^2^) compared with the conventional sequence (nominal resolution = 1.9 × 1.9 mm^2^), suggesting that the proposed sequence achieves higher spatial resolution in vivo. Furthermore, the sharpness index measured in a phantom was equivalent for the proposed sequence and a fully sampled acquisition at the same nominal resolution (1.4 × 1.4 mm^2^), suggesting equivalent achieved resolution when confounding factors of motion and partial volume-effects are removed.^52^

The temporal regularization incorporated in the CS reconstruction framework results in sensitivity of this sequence to respiratory motion, which is manifested in the images as ghosting artifacts. In this study, we maintained the regularization parameter at a modest level, which has been evaluated as optimal for linear undersampling in previous work.^33^ Higher sensitivity to artifacts was observed in non-breath-hold images acquired before and after the first pass of the contrast agent. However, these non-breath-hold images did not have any noticeable effect on the image quality of breath-hold images acquired during the first pass, which are the most relevant for clinical evaluation. One patient was unable to hold their breath, and the resultant images suffered from the presence of ghosting artifacts (Figure 6).This affected the overall image quality score for this patient, but the images were still recorded as diagnostic. No additional coaching in breath-holding was performed beyond the standard guidance given at the start of a CMR examination. Additional coaching may lead to improved overall image quality results with this sequence. It is also noted that breath-holding capabilities (among other physiological factors) may be affected under stress conditions. Prospective slice tracking^53,54^ and the integration of motion estimates in the reconstruction process^55^ may improve the robustness of the sequence to motion and will be investigated in future work. Temporal regularization could also result in a degree of temporal smoothing of the dynamic contrast changes. Although this effect is difficult to quantify in vivo, it should have been minimal, as no differences in upslope index and contrast ratio measurements were observed between the proposed and conventional sequences.

Quantitative assessment of SNR is challenging in this study due to the combination of parallel imaging and CS reconstruction, which results in a heterogeneous noise distribution throughout the image.^56^ Nevertheless, SNR is an important metric in the perception of CMR perfusion images, which is why we included a qualitative assessment of perceived SNR to capture any perceived difference in this metric between the two sequences.

The algorithm for the pseudorandom undersampling scheme used in this study prevents jumps from upper to lower k-space. The k-space indices randomly selected from each bin are also sorted in ascending order to limit k-space jumps. It is acknowledged that without any further optimization, k-space trajectories generated across dynamics could include varying amplitudes of k-space jumps (Supporting Information Figure S2), which could result in increased sensitivity to eddy current artifacts in some dynamics. However, unbalancing of the slice gradient has been shown previously as a mechanism to reduce eddy current artifacts in bSSFP sequences,^49^ which could be an added benefit of the GC-LOLA technique, as previously acknowledged.^32^ The intraslice dephasing resulting from the GC-LOLA gradient unbalancing in our in vivo study was between ±20° and ±30°. According to the results from a previous study,^57^ this level of dephasing should significantly reduce eddy current artifacts, without significantly affecting the SNR of the images. In practice, we found that eddy current artifacts were not noticeable in the reconstructed images and did not result in impaired image quality, as reflected in the results of the phantom and patient studies.

A limitation of the sequence is the time required for inline CS reconstruction using a CPU implementation (>30 minutes per acquisition). However, this could be significantly accelerated using a GPU implementation that can reduce the reconstruction time to a clinically acceptable time.

There are some limitations associated with the study. In the patient study, the proposed SMS-bSSFP CS with pseudorandom undersampling was compared with a conventional perfusion protocol but not with SMS-bSSFP CS with linear undersampling.^33^ This choice was motivated by the difficulties to perform three contrast injections per patient and associated increased scan time. Therefore, a conventional perfusion protocol was chosen as a reference in patients, as it is more clinically and commercially available. Nevertheless, the superiority of the proposed pseudorandom undersampling scheme over linear undersampling for SMS-bSSFP CS is demonstrated in the phantom study. A 10-minute delay between the two perfusion scans was used, which results in some residual gadolinium remaining in the myocardium before the administration of the second bolus. However, this delay time has been used widely for CMR perfusion studies,^3^ and we randomized the order of the sequence to mitigate this effect. The study was carried out in a limited number of patients referred for standard CMR imaging, and all acquisitions were performed at rest, although the overall acquisition time (540 ms/heartbeat) was defined to be compatible with stress conditions. Therefore, further validation of the sequence in a large group of patients with suspected coronary artery disease and under stress conditions is warranted.

## 6 Conclusions

A reordering algorithm has been developed to perform pseudorandom k-space undersampling that is compatible with SMS-bSSFP and CS reconstruction. This study has demonstrated that the high acceleration achievable with this technique enables increased spatial resolution and myocardial coverage of 1.5T CMR perfusion imaging, with an associated increase in the number of diagnostic segments and perceived SNR with no difference in image quality. Further validation of this sequence in patients with coronary artery disease is warranted.

## Supplementary Material

Supplementary Material

## Figures and Tables

**Table 1:** Sharpness indices (mm^-1^) measured in a phantom using fully sampled single-band acquisitions and the proposed SMS-CS sequence

	1.4 mm^2^	1.5 mm^2^	1.6 mm^2^	1.7 mm^2^	1.9 mm^2^
Fully sampled	0.71 ± 0.02	0.66 ± 0.01	0.61 ± 0.01	0.57 ± 0.02	0.50 ± 0.00
SMS-CS	0.74 ± 0.03	0.65 ± 0.01			
