# Supplementary material for: Combined simultaneous multislice bSSFP and compressed sensing for first-pass myocardial perfusion at 1.5 T with high spatial resolution and coverage
Source: Magn Reson Med. Author manuscript; Available in PMC 2021 Jul 26. (PMC7611375; doi:10.1002/mrm.28345)
Supplement: Supplementary Material [file EMS128774-supplement-Supplementary_Material.pdf]

SUPPORTING INFORMATION

Additional Supporting Information may be found online in the Supporting Information section.

FIGURE S1 Subtraction images produced by subtracting each image series acquired with an in-plane acceleration factor of 3.5 and above from the corresponding series acquired with an in-plane acceleration factor of 2.5. Resultant subtracted signal intensities are normalized to 25% of the maximum signal of the reference image (S_{max}). Normalized RMS errors are presented as a percentage of the signal range of the reference image (in-plane acceleration = 2.5)

FIGURE S2 Example pseudorandom k-space trajectories for the proposed undersampling scheme. A, k-Space trajectory of the dynamic with the largest maximum absolute jump in phase-encoding direction (K_y). B, Absolute jumps in K_y, plotted as a function of TR for the k-space trajectory plotted in (A). C, k-Space trajectory of the dynamic with the smallest maximum absolute jump in phase-encoding direction (K_y). D, Absolute jumps in K_y, plotted as a function of TR for k-space trajectory plotted in (C)
